# Dioscin protects against chronic prostatitis through the TLR4/NF-κB pathway

**DOI:** 10.1515/med-2024-1036

**Published:** 2024-09-13

**Authors:** Yan Long, Xiaodong Ge, Liangliang Ma, Junhua Guo, Yong Zhu

**Affiliations:** Department of Andrology, Yancheng TCM Hospital Affiliated to Nanjing University of Chinese Medicine, Yancheng, 224001, China; Department of Andrology, Yancheng TCM Hospital Affiliated to Nanjing University of Chinese Medicine, No. 53 Renmin North Road, Yancheng, 224001, China

**Keywords:** dioscin, chronic prostatitis, TLR4/NF-κB pathway

## Abstract

This study aimed to elucidate the effects and potential mechanisms of dioscin on chronic prostatitis (CP) *in vivo* and *in vitro*. CP models were constructed *in vivo* and *in vitro* and treated with different concentrations of dioscin. Hematoxylin and eosin staining was used to investigate the morphology of the prostate tissues. The concentration of inflammatory factors in prostate tissues was determined by enzyme-linked immunosorbent assay. The release of reactive oxygen species, malondialdehyde, superoxide dismutase, and catalase was measured using detection kits. P69 cell proliferation was assessed by 3-(4, 5-dimethylthiazol-2-yl)-2,5-diphenyl tetrazolium bromide. Furthermore, the activity of the TLR4/NF-κB signaling pathway was determined by quantitative reverse transcriptase polymerase chain reaction or Western blot assay. Histopathological data suggested that dioscin exerted protective effects against prostate morphological changes. Dioscin inhibits inflammatory cytokines and oxidative stress (OS) in prostate tissues in a concentration-dependent manner. Moreover, dioscin notably inhibited the activation of the TLR4/NF-κB signaling pathway in CP rats. *In vitro*, dioscin remarkably reduced lipopolysaccharide-induced P69 proliferation, inflammation, OS, and TLR4/NF-κB pathway activation in a dose-dependent manner. In conclusion, dioscin exerts a protective effect in CP by decreasing the inflammatory response and OS through the TLR4/NF-κB pathways. Our findings provide a novel latent therapy for dioscin for the treatment and prevention of CP.

## 1 Introduction

Chronic prostatitis (CP), a common urogenital disease with complex and diverse symptoms, mainly occurs in males aged 20–50 years [1,2]. The etiology of CP is complex and studies have shown that it may be caused by primary or secondary damage to the prostate and its surrounding tissues, organs, muscles, and nerves [3]. Due to the unclear and complex etiology of CP, unimodal therapy is largely unsuccessful. Therefore, CP treatment has transitioned to multimodal methods, including both pharmacological (such as antibiotics and alpha-blockers) and non-pharmacological (pelvic floor physical therapy, acupuncture, and electroacupuncture) treatments [4].

Inflammation, abnormal immune responses, oxidative stress (OS), damage, and endocrine disorders are closely associated with the occurrence of CP.

OS is a result of the imbalance between reactive oxygen species (ROS) and antioxidants in the body that can cause tissue damage and has a significant involvement in the pathogenesis of CP [5]. ROS plays an important role in cell signaling and homeostasis under normal conditions; however, imbalanced ROS interact with lipids, proteins, carbohydrates, and nucleic acids [6,7]. Continuous exposure of prostate tissue to inflammation can lead to a strong release of ROS, resulting in changes in protein structure and function [8]. Qian et al. revealed that resveratrol attenuates prostatic inflammation in rats with estradiol-induced CP [9]. Moreover, Zhao et al. suggested that lycopene attenuates CP/chronic pelvic pain syndrome by inhibiting OS and inflammation via the interaction of NF-κB, MAPKs, and Nrf2 signaling pathways in rats [10]. Currently, antibiotics, alpha-blockers, and antiphlogistics are commonly used for treating CP. Although the pathogenesis of CP is well understood, effective treatments are lacking [11]. Medications can temporarily relieve some symptoms; however, their long-term effects are not ideal. Therefore, active exploration of effective measures to prevent and treat CP has become a research hotspot. Dioscin is a natural steroidal saponin extracted from plants of the *Dioscorea* genus [12]. Research has shown that dioscin exerts antioxidant, anti-inflammatory, and anticancer effects in various diseases. Zhang et al. revealed that dioscin alleviates myocardial infarction injury by regulating BMP4/NOX1-mediated OS and inflammation [13]. Moreover, Xu et al. found that dioscin attenuates LPS-induced inflammatory myocardial injury via OS-related pathways [14]. Zhu et al. suggested that dioscin enhances osteoblastic cell differentiation and proliferation by inhibiting cell autophagy via the ASPP2/NF-κB pathway [15]. However, the effects and molecular mechanisms of the action of dioscin on CP remain unclear. Studies have shown that inflammation, abnormal immune responses, and OS damage are closely associated with CP [16]. LPS, a component of the outer wall of gram-negative bacteria, often acts on toll-like receptor 4 (TLR4) on the surface of epithelial cell membranes to induce an inflammatory response [17]. TLR4 is involved in the nonspecific immunity and intracellular signal transduction through the NF-κB pathway [18]. Reports have suggested that inhibition of the TLR4/NF-κB pathway may reduce LPS-induced inflammation [19]. In recent years, LPS-induced prostate epithelial cells have been used for *in vitro* research on CP [20,21]. Therefore, the inhibition of inflammation by regulating the TLR4/NF-κB signaling pathway may be an effective treatment method for CP.

## 2 Materials and methods

### 2.1 Animals

Male standard deviation (SD) rats (6–8 weeks old) were purchased from Animal Biotech Industries, housed in a temperature-controlled room with a 12-hour light/dark cycle and 60–65% humidity and allowed to feed freely. All animal experiments were approved by the Animal Care and Use Committee of the Yancheng TCM Hospital Affiliated to Nanjing University of Chinese Medicine (approval number: DW220803) and conducted according to accepted protocols.

### 2.2 Cell culture and treatment

P69 cells were purchased from American Type Culture Collection (ATCC) and seeded in Roswell Park Memorial Institute medium (Gibco, MD, USA) containing 10% fetal bovine serum (Invitrogen) and 1% penicillin–streptomycin and maintained at 37°C with 5% CO_2_ in an incubator. P69 cells were treated with 0.02 μg/mL LPS for 24 h to conduct subsequent experiments.

### 2.3 Establishment of CP model

Male SD rats were divided into five groups (*n* = 10): control, model, model + dioscin (15 mg/kg), model + dioscin (30 mg/kg), and model + dioscin (60 mg/kg) [22]. After anesthesia with ether, the abdominal skin was cut open to expose the prostate. Then, rats were injected twice with 3% carrageenan in the left and right ventral lobes of the prostate to establish a CP model. Rats in the control group were injected with a 0.9% sodium chloride solution. The CP model was successfully established after 7 days, and high, medium, and low doses of dioscin were administered by gavage. After 4 weeks, the prostate tissues of the rats in each group were removed, and the rats were sacrificed by peeling off their necks.

### 2.4 Hematoxylin and eosin staining

Prostate lateral lobe tissues were embedded in paraffin and cut into 5-μm-thick tissue blocks. Paraffin blocks were dewaxed, hydrated, and stained with hematoxylin for 3–10 min. To remove floating colors, the tissue blocks were washed with tap water and differentiated using 1% hydrochloric acid. After the tap water turned blue, the tissue blocks were stained with eosin at room temperature for 1–3 min. The tissue blocks were dehydrated, made transparent in anhydrous ethanol and xylene, and sealed with neutral gum. The morphology of the prostate tissue was observed under a microscope. Experiments were conducted independently three times.

### 2.5 Enzyme-linked immunosorbent assay (ELISA)

After treatment, prostate tissues were thoroughly ground with a homogenizer and centrifuged, and the supernatant was collected. The samples were cultured in a 96-well plate and HRP-conjugate reagent was added to each well. Then, the levels of inflammatory factors (interleukin-1beta [IL-1β], interleukin-6 [IL-6], and tumor necrosis factor alpha [TNF-α]) in samples were detected using ELISA kits (ELK Biotechnology) following the instructions. The optical density (OD) of each well at 450 nm was measured on a Microplate reader (SMR16.1, USCNK), according to the manufacturer’s instructions. Experiments were conducted independently three times.

### 2.6 ROS assay

Prostate tissues were subjected to ROS immunofluorescence staining. First, the tissues were circled with a histochemical pen to prevent the incubating liquid from flowing away. ROS-DHE solution (KGAF019, KeyGEN) was added and incubated in the dark for 30 min. After three washes with phosphate buffer solution (PBS), 4′,6-diamidino-2-phenylindole solution (D8417-1MG; Sigma) was added, and the cells were incubated at room temperature for 20–30 min in the dark. The sections were washed three times with PBS and photographed under a fluorescence microscope (IX51; OLYMPUS) as soon as possible. Experiments were conducted independently three times.

### 2.7 Measurement of malondialdehyde (MDA), superoxide dismutase (SOD), and catalase (CAT) levels in prostate tissues

After treatment, the MDA content and SOD and CAT activities in prostate tissues were checked using a Lipid Peroxidation (MDA) Assay Kit, Total Superoxide Dismutase Assay Kit, and Catalase Assay Kit (Nanjing Jiancheng, Nanjing, China), respectively, according to the manufacturer’s instructions. Experiments were conducted independently three times.

### 2.8 Measurement of ROS generation, MDA content, and SOD and CAT activities in P69 cells

After treatment, the ROS generation, MDA content, and SOD and CAT activities in P69 cells were checked using a Reactive Oxygen Species Assay Kit, Lipid Peroxidation (MDA) Assay Kit, Total Superoxide Dismutase Assay Kit, and Catalase Assay Kit (Beyotime, Shanghai, China), respectively, according to the manufacturer’s instructions. Experiments were conducted independently three times.

### 2.9 Western blot assay

Total protein from prostate tissues and P69 cells were prepared and lysed in the radioimmunoprecipitation assay lysis buffer (AS1004, Aspen Biosciences, Murrieta, CA, USA) and protease inhibitor cocktail (04693159001, Roche, Basel, Switzerland) for 30 min at 4°C. The lysate was centrifuged for 5 min at 12,000 rpm and 4°C, the upper supernatant was collected, and the protein was separated by 10% sodium dodecyl sulfate-polyacrylamide gel electrophoresis and transferred onto polyvinylidene fluoride membranes (IPVH00010; Millipore, Burlington, MA, USA). The membranes were blocked with 5% nonfat milk for 60 min, washed three times in TBST buffer, and incubated with specific primary antibodies against TLR4 (19811-1-AP, Wuhan Sanying, 1:1,000), p-p65 (#3033, CST, 1:1,000), p65 (#8242, CST, 1:3,000), or GAPDH (ab181602, Abcam, 1:1,000) at 4°C overnight. The membranes were then washed three times with TBST buffer and incubated with secondary antibodies for 30 min. Protein bands were detected using an enhanced chemiluminescence kit (AS1059, Aspen) and quantified using AlphaEaseFC software. Experiments were conducted independently three times.

### 2.10 Quantitative reverse transcriptase polymerase chain reaction (qRT-PCR) assay

Total RNA was extracted from prostate tissues and P69 cells using TRIpure Total RNA Extraction Reagent (EP013; ELK Biotechnology, Wuhan, China), following the manufacturer’s instructions. Total RNA was eluted and stored at −80°C. Then, cDNA was synthesized using the EntiLink™ 1st Strand cDNA Synthesis Super Mix (EQ031, ELK Biotechnology). qRT-PCR was performed using the EnTurbo™ SYBR Green PCR SuperMix (EQ001, ELK Biotechnology). The level of TLR4 was determined using the QuantStudio 6 Flex System (Life Technologies, Carlsbad, CA, USA). The primer sequences are listed in Table 1. Experiments were conducted independently three times.

**Table 1:** Primer sequences for PCR

Gene name	Sequences (5′–3′)	
R-GAPDH	Sense	GCCAAGGTCATCCATGACAAC
	Antisense	GTGGATGCAGGGATGATGTTC
R-TLR4	Sense	CCCAATTGACTCCATTCAAGC
	Antisense	CCTGAACTCATCAATGCTCACAT
H-GAPDH	Sense	CATCATCCCTGCCTCTACTGG
	Antisense	GTGGGTGTCGCTGTTGAAGTC
H-TLR4	Sense	GGATGAGGACTGGGTAAGGAAT
	Antisense	AATGAAGATGATACCAGCACGAC

### 2.11 3-(4,5-Dimethylthiazol-2-yl)-2,5-diphenyl tetrazolium bromide (MTT) assay

After treatment, P69 cells were implanted into 96-well plates and cultured for 24 h at 37°C. Then, cells were treated with 10 μL MTT solution and continuously incubated for a further 4 h. Afterwards, the solution was removed and 100 μL dimethyl sulfoxide was added to each well to solubilize the formazan product. Finally, the OD at 570 nm was measured by a microplate reader (BMG Labtech, Ortenberg, Germany) following the manufacturer’s protocol. Experiments were conducted independently three times.

### 2.12 Statistical analysis

All results were presented as the mean ± SD and analyzed using GraphPad Prism 6.0. Differences among groups were estimated using one-way analysis of variance with Tukey’s post hoc test or Student’s *t*-test. *P < 0.05 and **P < 0.01 indicated statistically significant differences.


**Ethical approval**: The experimental protocols were approved by Ethical Committee of the Experimental Animal Center of Yancheng TCM Hospital Affiliated to Nanjing University of Chinese Medicine according to the National Institutes of Health Guide for the Care and Use of Laboratory Animals (approval number: DW220803).

## 3 Results

### 3.1 Dioscin remarkably relieved prostate tissue damage in CP rats

**Figure 1:**
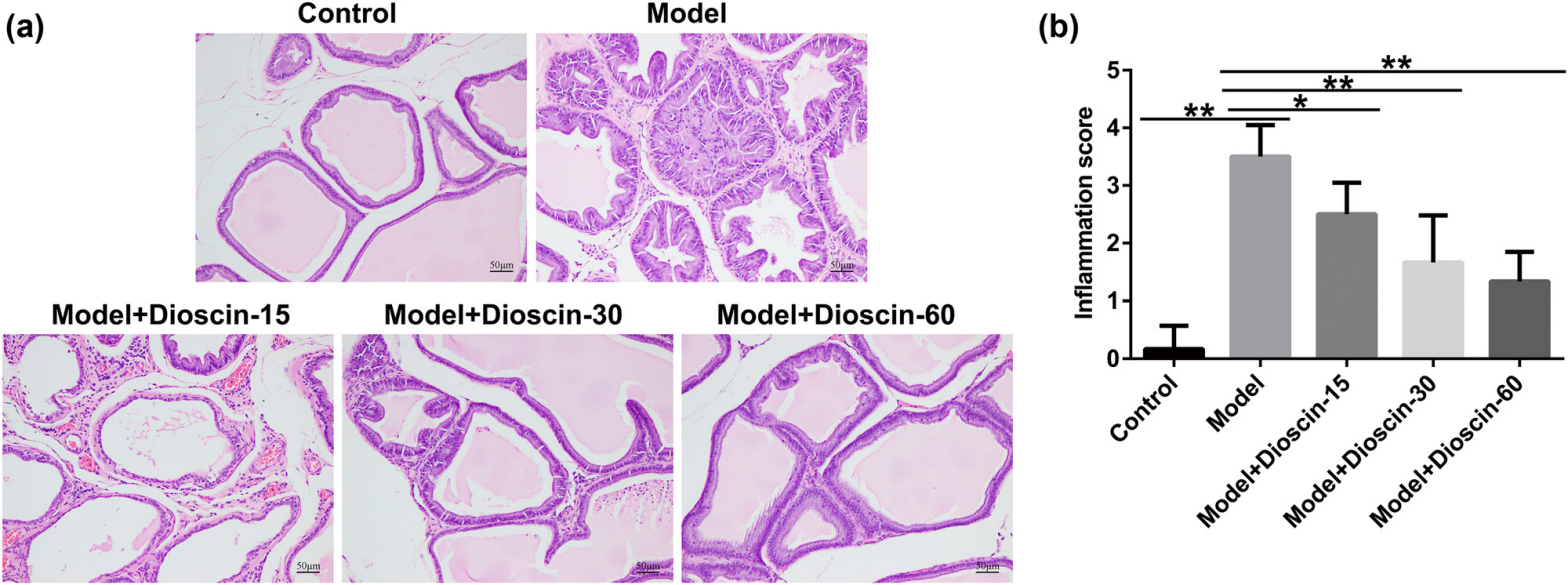
Dioscin treatment attenuates prostate damage in CP rats. CP rats were treated with different concentrations of dioscin and divided into five groups: control, model, model + 15 mg/kg dioscin, model + 30 mg/kg dioscin, and model + 60 mg/kg dioscin. (a) Prostate tissue morphology was assessed after HE staining. (b) Inflammation scores of the prostate tissue. *n* = 10. *P < 0.05, **P < 0.01. Experiments were conducted independently three times.

In the carrageenan-induced CP model, severe and stable lymphocyte infiltration, reduced acinar diameter, glandular cavity expansion, and interstitial edema were observed, confirming the successful establishment of the model. The CP rats were then orally administered low, medium, and high doses of dioscin (15, 30, and 60 mg/kg, respectively). After 28 days of dioscin administration, the gland cavity structure of the prostate tissue was repaired, the infiltration of inflammatory cells in the glandular lumen and interstitial space was suppressed, and the proliferation of fibrocytes and blood vessels was inhibited in a dose-dependent manner. This improvement was most pronounced in the high-dose dioscin group. This confirmed the function of dioscin in relieving CP from a histomorphological perspective (Figure 1a). Moreover, compared with the control group, the inflammation score of the prostate tissue was significantly increased in the model group, and this increase was decreased by dioscin in a dose-dependent manner (Figure 1b). Our findings revealed that dioscin significantly reduced prostate tissue damage in rats with CP.

### 3.2 Dioscin improved inflammatory response in CP rats

**Figure 2:**
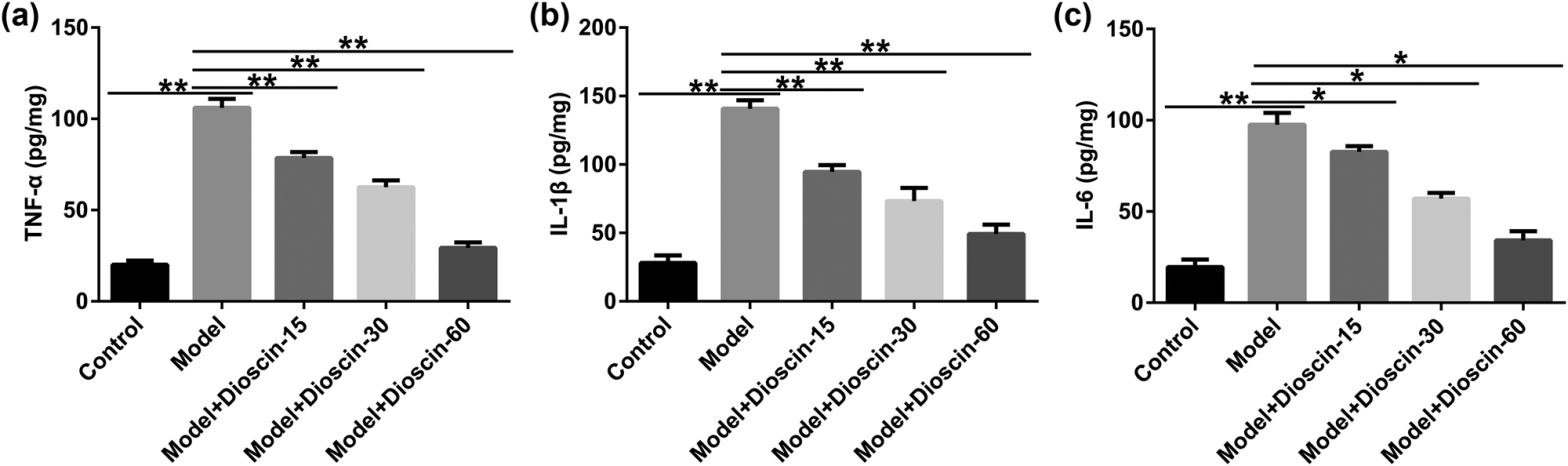
Effects of dioscin on the inflammatory response in prostate tissues of rats. The concentration of TNF-α, IL-1β, and IL-6 in the prostate tissues of rats (a)–(c) were assessed by ELISA. *P < 0.05, **P < 0.01. Experiments were conducted independently three times.

In the CP model, the levels of inflammatory cytokines in rat prostate tissues were measured using ELISA. TNF-α, IL-1β, and IL-6 expression in rat prostate tissues (Figure 2a–c) were higher in the model groups than those in the control group, and in all dioscin groups, dioscin inhibited promotion in a dose-dependent manner. These results showed that dioscin repressed the inflammatory response in CP.

### 3.3 Dioscin notably depressed OS in CP rats

**Figure 3:**
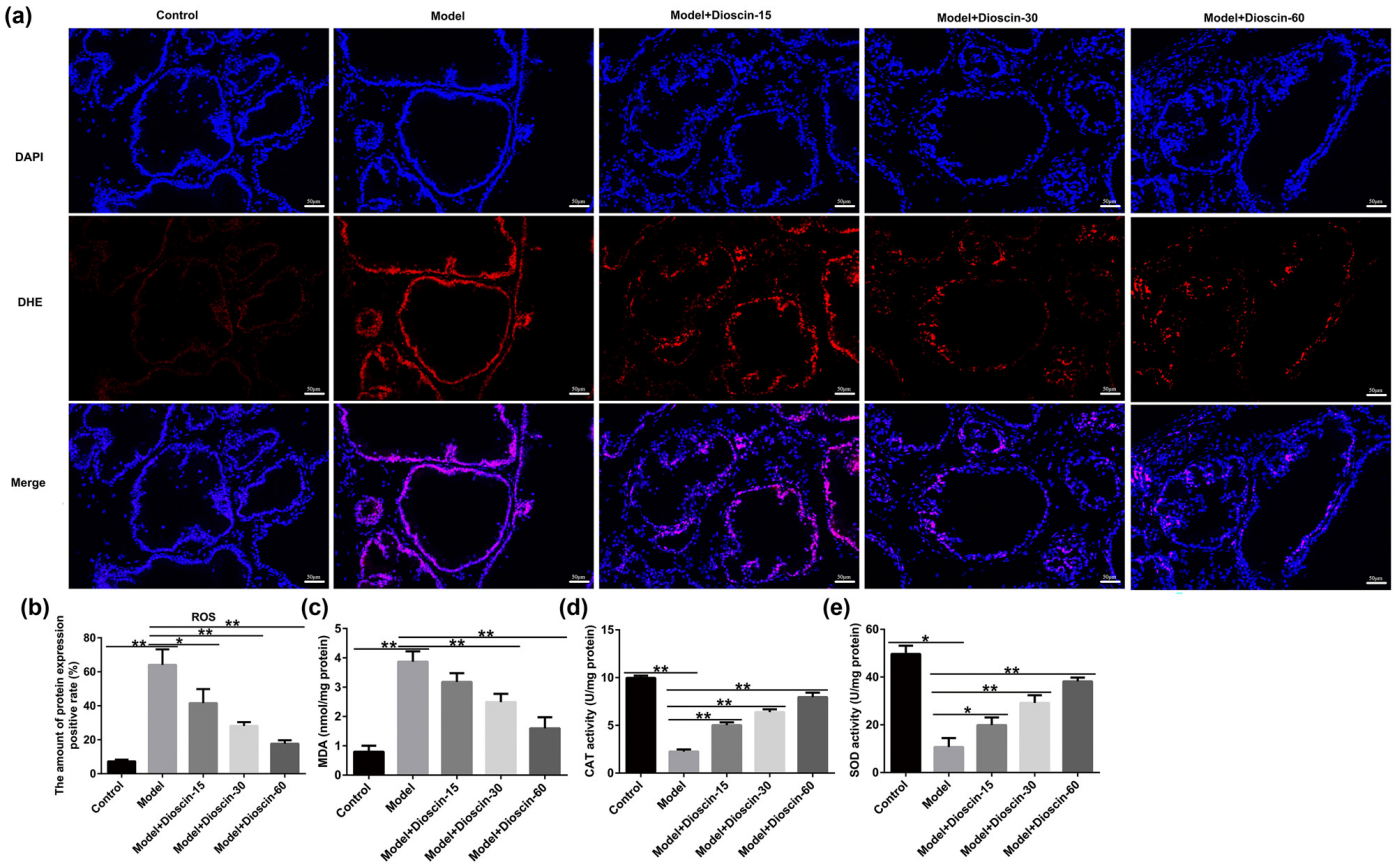
Effects of dioscin on oxidative stress in prostate tissues of rats. (a) and (b) The ROS levels were measured (magnification, ×200). (c) MDA concentration was measured. The activities of SOD (d) and CAT (e) in the prostate tissues were detected. *P < 0.05, **P < 0.01. Experiments were conducted independently three times.

Previous studies demonstrated that OS is closely associated with CP. Thus, we investigated whether dioscin is involved in OS in CP by measuring ROS, MDA, SOD, and CAT levels. As shown in Figure 3a–e, we observed higher ROS production and MDA levels, as well as lower SOD and CAT activities in the model group than in the control group. Dioscin treatment markedly suppressed ROS production and MDA levels and upregulated the activities of SOD and CAT in a dose-dependent manner, suggesting that dioscin suppresses OS and inflammation in CP rats.

### 3.4 Dioscin notably inhibited the activation of TLR4/NF-κB signaling pathway in CP rats

**Figure 4:**
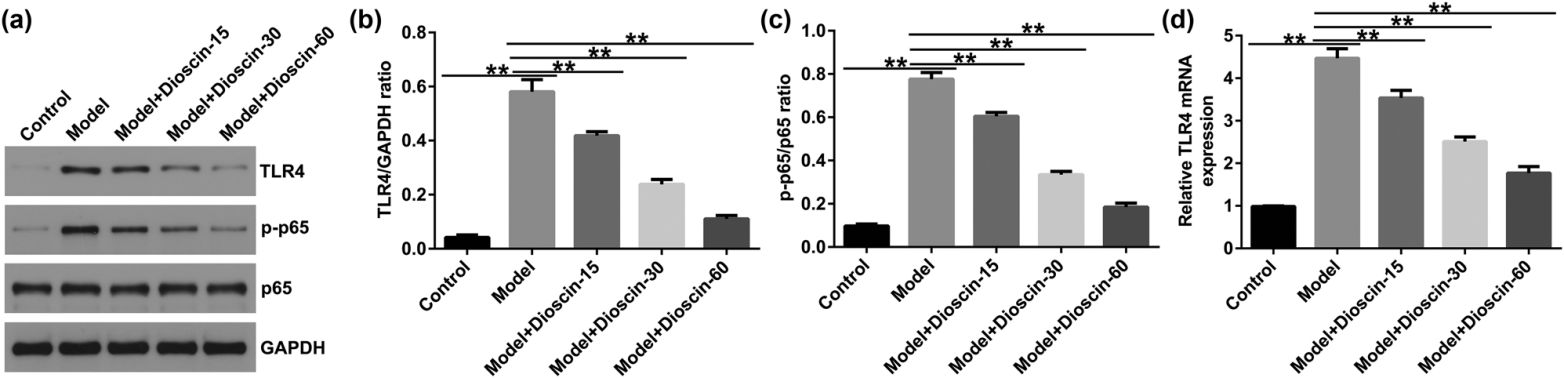
Effect of dioscin on TLR4/NF-κB pathway in prostate tissues of rats. (a) Western blotting analysis of TLR4 and p-p65 expression. (b) Quantification of the TLR4/GAPDH value. (c) Quantification of p-p65/p65 value. (d) qRT-PCR analysis of TLR4 mRNA levels. *P < 0.05, **P < 0.01 vs control. Experiments were conducted independently three times.

The TLR4/NF-κB signaling pathway is thought to be involved in the inflammatory response. To further expound the mechanism of dioscin in CP, its effect on TLR4/NF-κB signaling was determined in prostate tissues of rats. As shown in Figure 4a–d, the expression of TLR4 and p-p65 in the model groups was enhanced, as opposed to the control groups, and was remarkably inhibited by dioscin. Moreover, an increased p-p65/p65 ratio value and mRNA levels of TLR4 were observed in the model groups, which were notably reduced after dioscin treatment. Our observations demonstrated that dioscin suppressed OS and inflammation in CP rats through TLR4/NF-κB signaling pathway.

### 3.5 Dioscin ameliorates inflammation and oxidative damage induced by LPS

**Figure 5:**
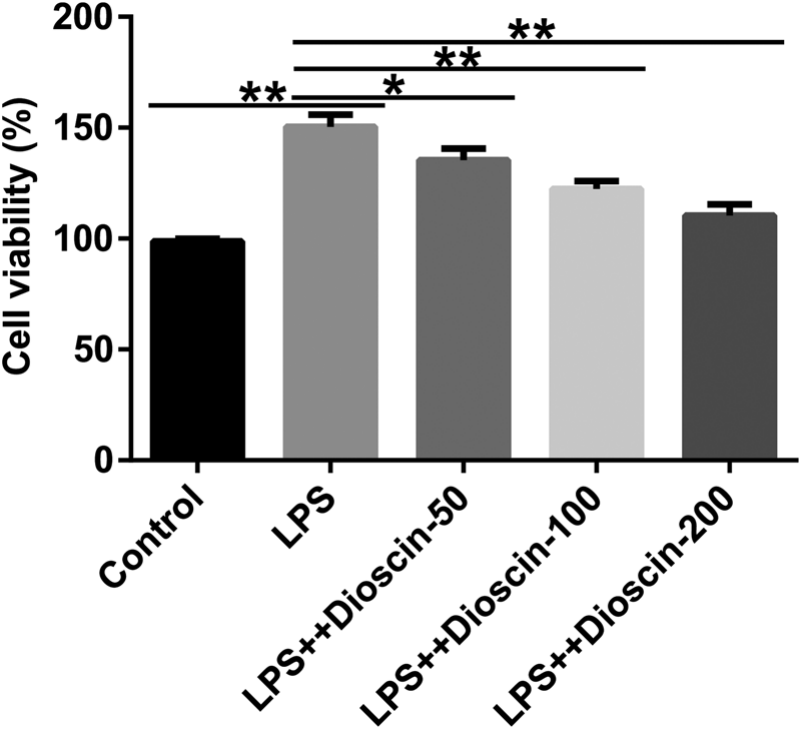
Effects of dioscin on LPS-induced P69 cell proliferation. P69 cells were induced using 0.02 μg/mL LPS and treated with different concentrations of dioscin. The cells were divided into five groups: control, model, model + 50 ng/mL dioscin, model + 100 ng/mL dioscin, and model + 200 ng/mL dioscin. The viability of P69 cells was determined using the MTT assay. *P < 0.05, **P < 0.01. Experiments were conducted independently three times.

**Figure 6:**
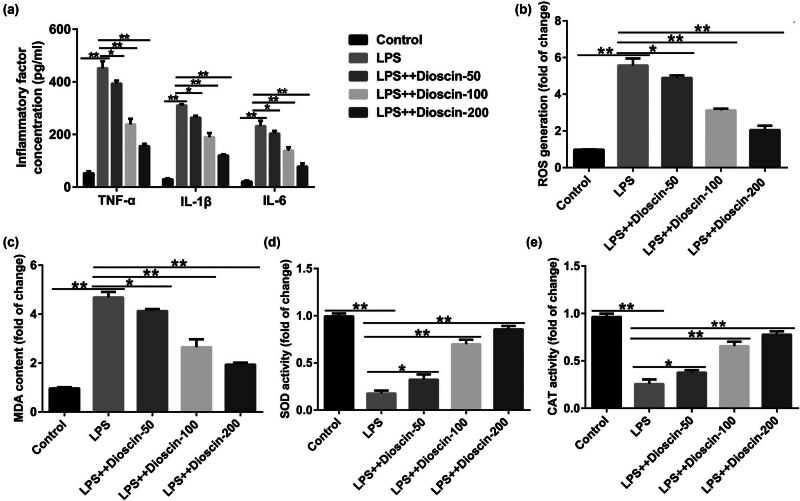
Effects of dioscin on inflammatory response and oxidative stress injury in LPS-induced P69 cells. P69 cells were induced using 0.02 μg/mL LPS and treated with different concentrations of dioscin. The cells were divided into five groups: control, model, model + 50 ng/mL dioscin, model + 100 ng/mL dioscin, and model + 200 ng/mL dioscin. (a) IL-1β, IL-6, and TNF-α concentration in LPS-induced P69 cells were assessed by ELISA. (b–e) ROS level, MDA concentration, and SOD and CAT activities were evaluated in P69 cells. *P < 0.05, **P < 0.01. Experiments were conducted independently three times.

Because inflammatory response and OS are associated with the pathological manifestations of CP, we determined the effects of dioscin on P69 cell viability, inflammatory factor release, and OS response. First, we determined the toxic effects of dioscin on P69 cells, and the data indicated that dioscin (50, 100, and 200 ng/mL) has no toxic side effects on P69 cells (Figure S1). Compared with the control group, dioscin (50, 100, and 200 ng/mL) markedly inhibited the LPS-induced proliferation of P69 cells in a dose-dependent manner (Figure 5). In addition, inflammatory cytokines (TNF-α, IL-1β, IL-6) were promoted (Figure 6a), ROS and MDA expression increased (Figure 6b and c), and SOD and CAT were suppressed (Figure 6d and e) in the LPS-induced P69 cells and were all reversed after dioscin treatment. Our findings revealed that dioscin relieved inflammatory response and oxidative damage in CP *in vitro*.

### 3.6 Dioscin suppressed the TLR4/NF-κB signaling pathway in P69 cells

**Figure 7:**
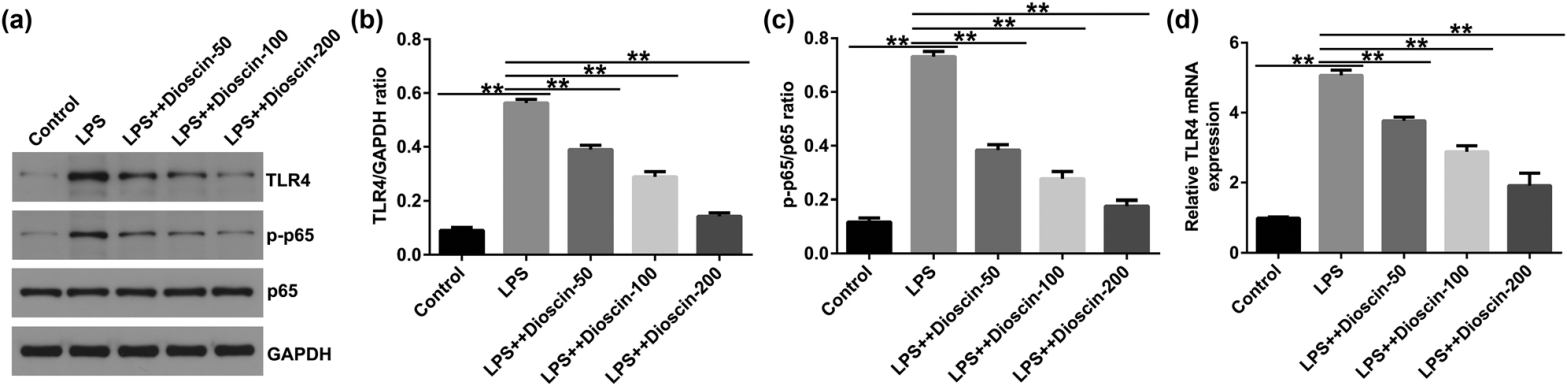
Effects of dioscin on TLR4/NF-κB pathway in LPS-induced P69 cells. (a) Western blotting analysis of TLR4 and p-p65 expression. (b) Quantification of the TLR4/GAPDH value. (c) Quantification of p-p65/p65 value. (d) qRT-PCR analysis of TLR4 mRNA levels. *P < 0.05, **P < 0.01. Experiments were conducted independently three times.

To explain the roles of the TLR4/NF-κB signaling in the dioscin treatment of P69 cells, we determined gene expression in the TLR4/NF-κB signaling pathway using Western blot and qRT-PCR analysis. Dioscin notably suppressed the activation of TLR4/NF-κB signaling, as confirmed by reduced TLR4 and p-p65 expression (Figure 7a and b), inhibited p-p65/p65 ratio (Figure 7c), and reduced TLR4 mRNA levels (Figure 7d) in P69 cells, compared with the model group. In summary, our findings demonstrated that dioscin protects against CP through the TLR4/NF-κB signaling pathway.

## 4 Discussion

CP is a common urological disease that mainly affects middle-aged men and is characterized by pelvic pain, irritating urination symptoms, and sexual dysfunction. Histologically, prostatitis is characterized by the presence of multinucleated and monocytic infiltrates in the stromal connective tissue surrounding the glands or duct [23,24]. The pathogenesis of CP is complex, and there is currently no effective treatment. Therefore, exploring effective measures to prevent CP and identifying promising strategies have become a research hotspot. Dioscin is a natural steroid saponin that promotes many biological processes, including cell proliferation, apoptosis, and OS [25]. Numerous studies have shown that dioscin exerts anti-inflammatory, anti-apoptotic, and antioxidant effects in various diseases. For instance, Xu et al. revealed that dioscin promotes liver regeneration by activating the Notch1/Jagged1 signaling pathway [26]. He et al. suggested that dioscin promoted prostate cancer cell apoptosis and inhibited cell invasion by increasing p-SHP1 levels and suppressing MAPK signaling [27]. However, little is known regarding the role of dioscin in CP. Our report was designed to explain the functions of dioscin in CP and clarify its molecular regulatory mechanisms.

First, 3% carrageenan was injected into the left and right ventral lobes of the prostate of SD rats to establish a rat model of CP. On the 7th day after treatment, the pathological changes were consistent with the characteristics of CP in Li’s model [28], with marked infiltration of lymphocytes, reduced acinar diameter, dilatation of the glandular cavity, and interstitial edema, demonstrating that the CP model had been successfully established. Nevertheless, after 28 days of dioscin, the pathological and histological changes in prostate tissues and lymphocyte infiltration caused by carrageenan were ameliorated, particularly in the high-dose group. Our data further suggested a significant increase in the inflammation score of prostate tissues in the model group compared with that in the control group, which was reduced by dioscin treatment, indicating the vital role of dioscin in improving prostate tissue damage in CP rats.

Increasing reports have demonstrated that suppressing the production of pro-inflammatory cytokines, such as IL-1β, IL-6, and TNF-α, can reduce the effect of prostatitis. Zhao et al. suggested the protective effects of dioscin against systemic inflammatory response syndrome via adjusting the TLR2/MyD88/NF-κB signaling pathway [29]. Moreover, Yao et al. demonstrated that dioscin reduces LPS-induced inflammatory liver injury by regulating the TLR4/MyD88 signaling pathway [30]. Consistent with these investigations, dioscin remarkably reduced the production of IL-1β, IL-6, and TNF-α *in vivo*. Furthermore, previous studies have shown that OS is related to the biological processes of dioscin.

All organisms exhibit homeostasis in oxidative and antioxidant processes, and OS occurs when the antioxidant capacity is weakened and oxides cannot be efficiently removed [31]. Accumulation of ROS and lipid peroxidation products (such as MDA) in the prostate tissue and restricted antioxidant substances (such as SOD and GSH) are considered the main mechanisms of prostatitis [32]. Based on previous studies, we assessed ROS, MDA, SOD, and CAT levels to determine whether dioscin exhibited antioxidative stress ability in CP. We observed higher ROS release and MDA levels, and lower SOD and CAT activities in the model groups than in the control group, which is similar to the findings of Jahan et al. [33], whereas we found the opposite results in a dose-dependent manner in the dioscin treatment groups. Our data indicate that OS is involved in the biological effects of dioscin.

After the activation of TLR family proteins, downstream signal transduction pathways may produce a complex signal cascade-amplifying response. NF-κB is a transcription factor consisting mainly of p50/p65 heterodimers that activate innate immune cells and the adaptive immune system via TLR4 [34]. Thus, NF-kB plays an important role in inflammatory and immune responses. TLR4 signaling induces a series of OS responses, including increased levels of ROS, NO, and MDA and decreased levels of SOD2, CAT, and other antioxidant enzymes [35]. Simultaneously, dioscin can alleviate Alzheimer’s disease and myocardial infarction injury through OS [13,36]. Consistent with these findings, the present study showed that dioscin alleviated inflammation and OS in prostate tissues in a concentration-dependent manner. Our results show that dioscin plays a protective role in CP by inhibiting the TLR4/NF-κB pathway, as confirmed by reduced TLR4 and p-p65 expression, as well as p-p65/p65 ratio. To further reveal the function of dioscin in the progression of CP, LPS was used to induce P69 cells to conduct a CP model *in vitro*. Our findings suggest that dioscin protects against CP by inhibiting P69 cell viability and suppressing the production of inflammatory cytokines and OS factors. Moreover, dioscin plays a protective role by regulating the TLR4/NF-κB pathway.

This study also has some limitations. First, this study did not conduct rescue experiments to demonstrate whether dioscin directly exerts its role in CP by inhibiting TLR4 expression. Besides, other pathways or genes may also be involved in the impact of dioscin on CP, which requires further exploration. Considering the effects of different dosages of dioscin over longer periods on CP could add depth to the findings. Moreover, clinical trials to test dioscin’s efficacy and safety in humans should be further studied. In the future, we will conduct in-depth research on these issues.

## 5 Conclusion

Dioscin can remarkably inhibit inflammatory cell infiltration in CP and reduce the inflammatory response and OS by regulating the TLR4/NF-κB pathway, which provides a basis for the treatment of CP with dioscin.

## Abbreviations

IL-1β: interleukin-1beta
IL-6: interleukin-6
TNF-α: tumor necrosis factor-alpha
ELISA: enzyme-linked immunosorbent assay
MTT: 3-(4,5-dimethylthiazol-2-yl)-2,5-diphenyl tetrazolium bromide
TLR4: Toll-like receptor 4
NF-κB: NF-kappa B
qRT-PCR: quantitative reverse transcriptase polymerase chain reaction
MAPKs: mitogen-activated protein kinases
Nrf2: NF-E2-related factor 2
BMP4: bone morphogenetic protein 4
NOX1: NADPH oxidase 1
LPS: lipopolysaccharide
PBS: phosphate buffer solution
DAPI: 4',6-diamidino-2-phenylindole
SDS-PAGE: sodium dodecyl sulfate-polyacrylamide gel electrophoresis
PVDF: polyvinylidene fluoride

## Supplementary Material

Supplementary Figure
