# Supplementary material for: Dioscin protects against chronic prostatitis through the TLR4/NF-κB pathway
Source: Open Med (Wars). 2024 Sep 13;19(1):20241036. doi: 10.1515/med-2024-1036 (PMC11406438; doi:10.1515/med-2024-1036)
Supplement: Supplementary Figure [file med-2024-1036-sm.pdf]

Supplementary material

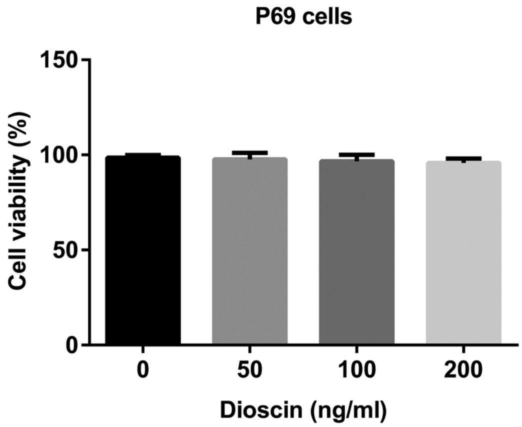

Figure S1: Toxic effects of dioscin on P69 cells. P69 cells were treated with different concentrations of dioscin for 24 h, then MTT was used to detect the cell viability. Experiments were conducted independently three times.
